# Assessing learning as a possible sign of consciousness in post-coma persons with minimal responsiveness

**DOI:** 10.3389/fnhum.2014.00025

**Published:** 2014-02-10

**Authors:** Giulio E. Lancioni, Andrea Bosco, Marta Olivetti Belardinelli, Nirbhay N. Singh, Mark F. O’Reilly, Jeff Sigafoos, Francesca Buonocunto, Jorge Navarro, Crocifissa Lanzilotti, Fiora D’Amico, Marina De Tommaso

**Affiliations:** ^1^Department of Neuroscience and Sense Organs, University of BariBari, Italy; ^2^Department of Educational Science, Psychology, Communication, University of BariBari, Italy; ^3^Department of Psychology, University “La Sapienza”Rome, Italy; ^4^Medical College of Georgia, Georgia Regents UniversityAugusta, GA, USA; ^5^University of Texas at Austin, AustinTX, USA; ^6^Victoria University of WellingtonWellington, New Zealand; ^7^S. Raffaele Rehabilitation and Care CentersCeglie and Alberobello, Italy

**Keywords:** learning, microswitches, minimally conscious state, vegetative state

## Abstract

A learning test procedure based on operant principles may be useful in the diagnosis (and eventually rehabilitation) of post-coma persons with minimal responsiveness. This study was aimed at extending the evaluation of such a procedure with seven participants who presented with very limited behavior and apparently severe disorders of consciousness. The procedure was evaluated through an ABACB design, in which A represented baseline phases without stimulation, B intervention phases with brief stimulation periods contingent on specific responses of the participants, and C a control phase in which stimulation was available all the time. Increased responding during the B phases, as opposed to the A and C phases, was taken to indicate learning and possibly a non-reflective expression of phenomenal consciousness. All participants were also evaluated with the coma recovery scale-revised (CRS-R) prior to the start of the learning test procedure and at the end of it. The results of the learning test showed that all participants had significantly higher responding levels during the B phases. The CRS-R scores suggested minimally conscious state for four of them prior to the learning test and for five of them after the completion of the learning test. The implications of the findings are discussed in terms of potential and time cost of the learning test.

## INTRODUCTION

Post-coma persons with extensive brain damage and minimal (or no apparent) responsiveness may present serious questions concerning the diagnosis of their state as well as the identification of the most suitable intervention strategy to promote their adaptive behavior (Lancioni et al., 2010a; Bruno et al., 2011; Gosseries et al., 2011; Fingelkurts et al., 2012, 2013; Laureys and Boly, 2012; Schnakers, 2012). Behavioral scales apparently represent the most frequently used tools for determining the persons’ general status and monitoring or predicting their possible progress (Cavinato et al., 2009; Whyte et al., 2009; Amantini et al., 2011; Lechinger et al., 2013; Ní Lochlainn et al., 2013). Behavioral scales, such as the coma recovery scale-revised (CRS-R) and the Wessex head injury matrix (WHIM), involve detailed assessment conditions that are considered adequate to identify relevant sensory and communication expressions or responses to objects easily missed in traditional bedside evaluations (Shiel et al., 2000; Kalmar and Giacino, 2005; Schnakers et al., 2009; Boly, 2011; Bruno et al., 2011; Doig and Lane-Brown, 2012; Godbolt et al., 2012; La Porta et al., 2013).

Neurophysiological strategies for assessing event-related brain potentials (P300 and mismatch negativity) are also frequently used with persons with severe disorders of consciousness, particularly persons with extensive levels of impairment and minimal (or no apparent) responsiveness (Fischer et al., 2008, 2010; Qin et al., 2008; Cavinato et al., 2009; Höller et al., 2011; Lehembre et al., 2012; King et al., 2013). The presence of recognizable P300 and mismatch negativity responses has been generally taken as a positive sign that attests to or predicts an evolution towards basic levels of awareness/consciousness (Wijnen et al., 2007; Vanhaudenhuyse et al., 2008; Fischer et al., 2010).

The use of neuroimaging techniques, such as functional magnetic resonance imaging (fMRI), represents the most sophisticated approach to enhance the assessment in cases of minimal (or no apparent) responsiveness (Owen and Coleman, 2008; Coleman and Pickard, 2011; Monti, 2012; Bodart et al., 2013; Harrison and Connolly, 2013; Owen, 2013). Such an approach has been instrumental to reach diagnostic answers for a number of controversial cases (e.g., Owen et al., 2006; Monti et al., 2010). It must also be pointed out, however, that its application can be a difficult methodological and practical challenge in most medical and care or rehabilitation contexts (Vul and Pashler, 2012; Harrison and Connolly, 2013; Mashour and Avidan, 2013).

Another approach for extending/enhancing the assessment of difficult cases might be the use of learning test procedures (Bekinschtein et al., 2009; Lancioni et al., 2009a; Kim et al., 2012; Monti, 2012). Two types of procedures have been reported as potentially successful tools. One was based on classical learning principles (Pierce and Cheney, 2008) and assessed the persons’ responding (i.e., eyelid closure) to a sound stimulus, which was paired with an air-puff (Bekinschtein et al., 2009). Learning consisted of responding immediately after the sound and independently of the air-puff. The other was based on operant learning principles (Pierce and Cheney, 2008) and assessed the persons’ frequency of a simple response (e.g., prolonged eyelid closure), which was followed by brief periods of positive environmental stimuli such as music. Learning consisted of an increase in the frequency of the response leading to stimulation (i.e., of an association between the response and the consequent stimulation). Signs of learning in each of the two procedures would (a) indicate an association between events (i.e., the specific sound and the air-puff in the first case and the response and the following stimulation in the second case), and (b) probably suggest a non-reflective level of phenomenal consciousness that could support a diagnosis of minimally conscious state (Bekinschtein et al., 2009; Bosco et al., 2009, 2010; Lancioni et al., 2009b, 2011).

In light of the above, the learning test procedures might be pointed out as an important aspect/supplement in the diagnostic process. The second (operant learning) procedure might have the relevant advantage of being suitable for rehabilitation intervention also (Machado and Korein, 2009; Lancioni et al., 2010a, 2012b). In fact, it might help the persons become more active (i.e., increase the frequency of the responses selected for them) to access their environment, enhance their level of sensory input, and show their preference for different stimuli and caregivers (Lancioni et al., 2010a, 2012b; Bagnato et al., 2013). The present study was aimed at providing confirmatory evidence on the applicability and impact/potential of the second procedure with a new group of participants that included seven post-coma persons with very limited behavior and apparently severe disorders of consciousness. All participants were evaluated with the CRS-R (i.e., the most widely used behavioral scale) prior to the start of the learning test procedure and at the end of it.

## METHODS

### PARTICIPANTS

The seven participants included two women (Carol and Doris) and five men (Alex, David, Neal, Damon, and Ray) who were in a neurological rehabilitation center or in medical care centers and presented with disorders of consciousness and pervasive motor impairment secondary to brain injury and coma. All participants also used a gastrostomy tube for enteral nutrition. Their families had provided informed consent for this study, which was approved by a scientific and ethics committee. Carol was 71 years old and had suffered a cardiac arrest with subsequent anoxic encephalopathy about 8 months prior to this study. Her coma lasted 4 weeks and then developed into a vegetative state, which had reportedly changed only minimally over time. Her CRS-R total score at the start of the learning test procedure was 7 (see **Table 1** for the scores on the subscales).

**Table 1:** Participants’ scores on the first (upper number) and second (lower number) application of the CRS-R.

Subscales	Participants						
	Carol	Doris	Alex	David	Neal	Damon	Ray
Arousal	2	2	2	2	2	2	1
	2	2	2	2	2	2	2
Oral/motor	1	1	2	0	1	0	0
	1	1	2	0	1	0	0
Motor	2	2	**5**	1	**3**	1	2
	2	2	**5**	1	**5**	2	2
Communication	0	0	0	0	0	0	0
	0	0	0	**1**	0	0	0
Visual	1	**2**	1	**2**	**2**	1	1
	**2**	**3**	1	**2**	**3**	1	1
Auditory	1	2	2	2	2	1	1
	2	2	2	2	2	1	1

Total score	7	9	12	7	10	5	5
	9	10	12	8	13	6	6

*Bold scores indicate minimally conscious state.*

Doris was 82 years old and had suffered aneurism rupture of the anterior communicating artery, with extended subarachnoid hemorrhage and coma about 4 months prior to this study. Angiographic arterial embolization was carried out with her. Her coma lasted 2 weeks and was replaced by a condition, which was soon considered compatible with the minimally conscious state. Her CRS-R total score at the start of the learning test procedure was 9 (see **Table 1**).

Alex was 34 years old, and had been involved in a road accident about 12 years prior to this study. The accident had resulted in diffuse axonal injury on bilateral temporal areas, right fronto-temporal region, and splenium of corpus callosum. His coma lasted 3 weeks and then developed into a vegetative state, which improved, over an unspecified time period, to a level compatible with the minimally conscious state. His CRS-R total score at the start of the learning test procedure was 12 (see **Table 1**).

David was 72 years old, and had suffered a left total anterior circulation stroke about 10 months prior to the study, with consequent left fronto-temporoparietal ischemic lesion. His coma lasted about 2 weeks and evolved into a vegetative state. This was reported to have apparently changed into a minimally conscious state about 2 months thereafter. His CRS-R total score at the start of the learning test procedure was 7 (see **Table 1**).

Neal was 27 years old and had suffered a severe fall about 5 months prior to the study. The fall had resulted in multiple cranial fractures with extended right hemispheric subarachnoid hemorrhage, right temporal subdural hematoma, and diffuse axonal injury of the right fronto-temporal region and splenium of corpus callosum. His coma lasted 3 weeks and then developed into a vegetative state, which lasted about 3 months and was apparently replaced by a minimally conscious state. His CRS-R total score at the start of the learning test procedure was 10 (see **Table 1**).

Damon was 43 years old, and had been involved in a road accident resulting in extended brain damage about 3 months prior to this study. He presented with diffuse axonal injury of the genu, body and splenium of corpus callosum, and mesencephalic region. His coma lasted 3 weeks and developed into a vegetative state that reportedly improved only with regard to the level of vigilance/alertness prior to the study. His CRS-R total score at the start of the learning test procedure was 5 (see **Table 1**).

Ray was 49 years old, and had suffered an extended left temporoparietal and ventricular hemorrhage with consequent hematoma about 6 months prior to this study. Following hematoma evacuation, he presented with a diffuse left fronto-temporoparietal necrotic lesion and hydrocephalus. Ray’s coma lasted about 4 weeks and was replaced by a vegetative state, which showed limited evolution over time. His CRS-R total score at the start of the learning test procedure was 5 (see **Table 1**).

### RESPONSES, MICROSWITCHES, AND STIMULI

During the sessions, Carol, Alex, David, Damon, and Ray lay in bed while Doris and Neal were either in bed or in a reclined position in a wheelchair. The responses were selected on the basis of their apparent suitability and ease (i.e., after direct and video observation of the participants’ repertoire). They consisted of (a) simple eyelid closure for David, (b) prolonged eyelid closure (i.e., a closure exceeding 0.5 s) for Carol and Alex, (c) repeated eyelid closure (i.e., two blinks within a 2-s interval) for Doris, (d) finger raising movement (i.e., a movement of the index finger from the right hand) for Neal, (e) movement of the thumb toward the index finger for Damon, and (f) forehead skin movement for Ray. The microswitch used for the eyelid closure responses was an optic sensor including an infrared light-emitting diode and a mini infrared-light detection unit (see Lancioni et al., 2012a). The microswitch was held through medical tape slightly above the participants’ right or left cheekbone (i.e., sufficiently distant from the eye so that it would not interfere with normal visual functioning). To ensure that the microswitch would detect eyelid closures, a small paper sticker was attached to the participants’ corresponding eyelid (Lancioni et al., 2012c). An optic microswitch was also used for Neal and Ray. For Neal, it was arranged slightly above the index finger of his right hand and was activated whenever the finger was raised (i.e., moved close to the microswitch). For Ray, it was attached to the forehead, together with a black mini sticker, and was activated as the forehead skin moved upward (i.e., as the microswitch pointing shifted from the sticker to the skin; Lancioni et al., 2013). A two-membrane small device was attached to the index finger of Damon’s right hand. It was activated via a small contact or pressure of the thumb (Lancioni et al., 2012a).

Microswitch activation triggered a computer system that activated a 10-s presentation of stimuli considered to be relevant/pleasant for the participants. The stimuli consisted of audio-visual inputs, namely, video clips of singing, comedy, films, and family events, which had been recommended by the participants’ families as preferred for the participants prior to their brain injury and coma. For Ray, who was reported to have unspecified visual and auditory impairment, video clips were combined with light body massages, namely, light stroking of his shoulders, arms, hands, and chest.

### EXPERIMENTAL CONDITIONS

The learning test procedure was carried out according to an ABACB sequence (a new A would be used after the C if this produced high responding, but such an event did not occur; Barlow et al., 2009). A represented baseline, B contingent stimulation, and C non-contingent stimulation. Sessions lasted 5 min and were implemented 4–11 times a day, depending on the participants’ schedule and wakefulness (i.e., a session was carried out only if the participant was awake). The test was completed in approximately 4 weeks. Prior to the beginning of the first A phase and after the completion of the second B phase, the participants were assessed through the CRS-R.

Data collection during the learning test consisted of recording the frequencies of the participants’ microswitch responses. Such recording was automatically carried out through the computer system. A new response would be recorded only if it occurred after an interval of 10 s from the previous one (i.e., after an interval equivalent to that covered by contingent stimulation episodes during the B phases). Response prompting (i.e., an air puff on the forehead or a tap on the finger) was available prior to the start of the sessions as well as during the sessions if periods of non-responding of 30–60 s occurred. The responses performed during the sessions through prompting were subtracted from the computer count (i.e., by the researchers in charge of the sessions). Agreement between researchers on recording these prompt-related responses (which could also be zero) was checked in 8–10 sessions for each participant and reported to be consistent.

#### Baseline (A) phases

During these phases, the participants were provided with their microswitch and the computer system. Responding was recorded, but no stimulation was available during the sessions. The baseline phases included four to eight sessions and ended if the response frequency of the last session was in line with or lower than that of the previous sessions.

#### Contingent stimulation (B) phases

Conditions were as in baseline except that stimulation (i.e., presentation of selected stimuli) occurred contingent on the participants’ responses. Each response was followed by a 10-s stimulation period. The two B phases included 45 and 30 sessions, respectively.

#### Non-contingent stimulation (C) phase

Conditions were as in the B phases except that stimulation (i.e., presentation of selected stimuli) occurred throughout the session. Thirty sessions were implemented.

#### Applications of the CRS-R

The CRS-R was applied prior to the start of the learning test procedure and at the end of it. The same two experts were responsible for the application of the scale and simultaneously present during the evaluations. The scoring was done on a consensus basis.

## RESULTS

**FIGURE 1:**
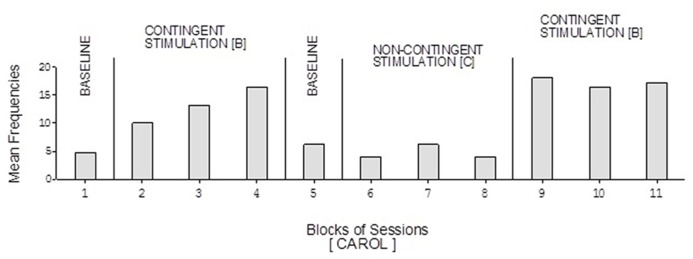
**Carol’s data during the different phases of the learning test.** The bars represent mean frequencies of responses per session over blocks of sessions. The blocks include six and eight sessions during the baseline phases, 15 sessions during the first B phase, and 10 sessions during the C phase and the second B phase.

**FIGURE 2:**
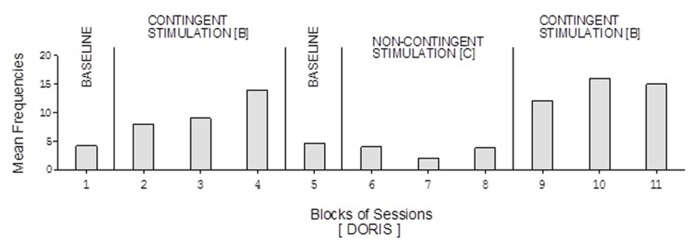
**Doris’ data plotted as in Figure 1 with variations on the number of baseline sessions (see text)**.

**FIGURE 3:**
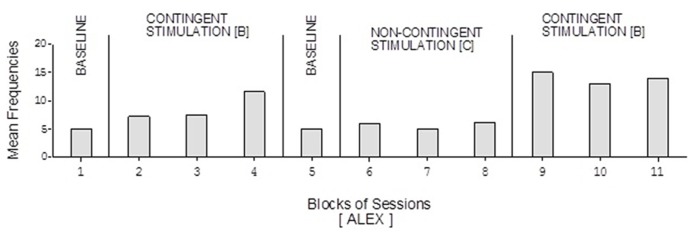
**Alex’s data plotted as in Figure 2**.

**FIGURE 4:**
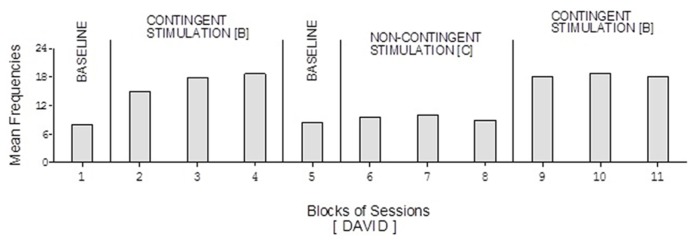
**David’s data plotted as in Figure 2**.

**FIGURE 5:**
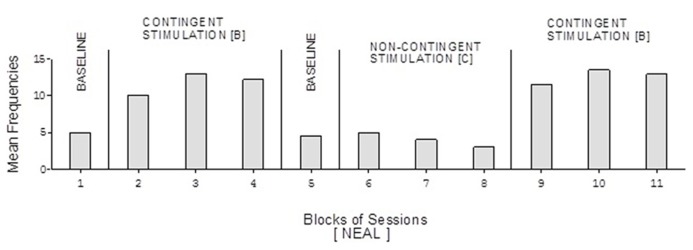
**Neal’s data plotted as in Figure 2**.

**FIGURE 6:**
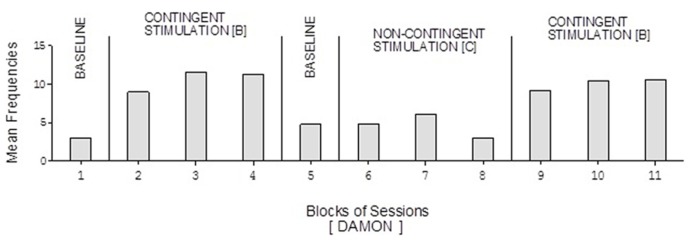
**Damon’s data plotted as in Figure 2**.

**FIGURE 7:**
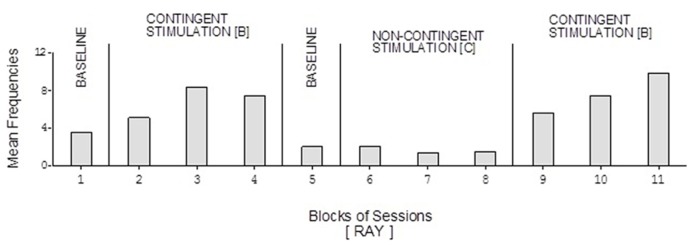
**Ray’s data plotted as in Figure 2**.

The participants’ frequencies of responses independent of prompts during the learning test (i.e., during the A, B, and C phases of the study) are summarized in **Figures 1–7**, respectively. The bars used within the figures represent mean frequencies of responses, per session, over blocks of sessions. The blocks include four to eight sessions during the baseline phases, 15 sessions during the first B phase, and 10 sessions during the C phase and the second B phase. Carol’s baseline phases showed mean frequencies of about five and six responses per session, respectively (**Figure 1**). Her two B phases showed mean frequencies of about 13 and 17 responses per session, respectively. Her non-contingent stimulation (C) phase showed a mean frequency of about five responses per session.

Doris’ baseline mean frequencies were below five per session (**Figure 2**). Her mean frequencies during the B phases exceeded 10 and 14 per session. Her mean frequency during the C phase was below four per session. Alex’s data were similar to those of Doris except for a slightly higher response frequency during the C phase (**Figure 3**). Neal’s data (**Figure 5**) were similar to those observed for Carol.

David’s baseline mean frequencies of responses were about eight per session (**Figure 4**). His mean frequencies during the B phases were about 17 and 18 per session. His mean frequency during the C phase was below 10 per session. Damon’s mean frequencies of responses per session were below five during the baseline phases, about 10 during the B phases, and again below five during the C phase (**Figure 6**). Finally, Ray’s mean frequencies per session were below four and two during the baseline phases, near seven and eight during the B phases, and about two during the C phase (**Figure 7**).

The Kolmogorov–Smirnov test (Siegel and Castellan, 1988) showed that the differences between the response frequencies of the A and B phases as well as of the B and C phases were statistically significant (*p* < 0.01) for all participants. These differences suggest that all participants showed signs of learning. Those signs might be taken to indicate some form of consciousness as suggested in the Section “Introduction.”

**Table 1** reports the participants’ scores on the single subscales of the CRS-R and their total scores during the first (upper numbers) and the second (lower numbers) application of the scale. The scores of the first evaluation suggest a diagnosis of (a) minimally conscious state for Doris, Alex, Neal, and David, and (b) vegetative state for Carol, Damon, and Ray. The scores of the second evaluation suggest a diagnosis of minimally conscious state for the four participants mentioned above and for Carol (due to her improvement in the visual subscale), and vegetative state for Damon and Ray. The data of the second CRS-R application indicate conformity with the learning test data of five of the seven participants.

## DISCUSSION

A learning test procedure relying on several specific responses, microswitches, and stimuli were effectively used with seven participants who displayed severe disorders of consciousness. The fact that learning was also visible with Damon and Ray whose CRS-R evaluations showed scores compatible with a diagnosis of vegetative state may be practically relevant. In fact, the learning test may be more sensitive than behavioral scales and help identify signs of consciousness when the general (sensory, motor, and communication) behaviors tapped by the scales do not yet suggest the presence of those signs. It might be that the CRS-R scores of those individuals increase over time and eventually get to match the learning evidence. Score increases and correspondence with the learning data, however, might not necessarily be expected in all cases (especially those with pervasive motor impairment or sensory disabilities; see Lancioni et al., 2010a).

The question of whether the learning evidence can be taken as a sign of consciousness has been tackled in previous studies (Bekinschtein et al., 2009; Bosco et al., 2009, 2010; Lancioni et al., 2009b, 2011). Their basic point has been that learning may be considered a non-reflective expression of phenomenal consciousness and, therefore, might support a diagnosis of minimally conscious state. Indeed, learning reflects the participant’s ability (a) to monitor/discriminate a specific response and the occurrence of environmental stimuli, (b) understand whether or not there is a relationship between them, and (c) adapt the response to the relationship or lack thereof (i.e., increasing the frequency of the response or leaving it unchanged; Pierce and Cheney, 2008; Catania, 2012). Such ability could not be discounted as irrelevant in terms of consciousness as well as in terms of adaptive responding (i.e., as evidence of the person dealing appropriately/meaningfully with the environment and its stimulation events; Spivey, 2007; Lancioni et al., 2010a; Monti, 2012; Bagnato et al., 2013).

The use of the learning test, as applied in this study (i.e., based on operant principles), may be considered rather costly in terms of time. In fact, it involves a relatively large number of sessions, which require a substantial investment on the side of staff personnel. While this observation cannot be downplayed, two considerations might be made about it. First, it is possible that the learning test might be carried out successfully with a smaller number of sessions for some participants, thus saving relevant staff time. Second, one could view the sessions not only as a costly assessment process but also as a positive stimulation period totally in line with the reported needs of persons with vegetative or minimally conscious state (Daveson, 2010; Lancioni et al., 2010a; Hirschberg and Giacino, 2011; Lotze et al., 2011; Di Stefano et al., 2012).

The use of stimuli such as light body massages (as done with Ray) may raise additional questions about the cost of the learning test based on operant principles. Indeed, those stimuli are to be administered by a staff person directly while the stimuli normally used (e.g., video clips of songs and films) are presented automatically by the technology available for the procedure. The reason for using body massages was the assumption that they could have greater reinforcing/motivating value than other stimuli (i.e., also in view of Ray’s presumed visual and auditory impairment). The use of pleasant (motivating) stimuli is undoubtedly critical to foster learning (Kazdin, 2001; Pierce and Cheney, 2008).

The use of a learning test procedure with the related technology for monitoring the participant’s responses (i.e., microswitches) and providing him or her stimulation (i.e., computer system) may be considered highly practical and functional also for rehabilitation purposes. In fact, this procedure does not simply allow the participant an increase in stimulation input (as it happens with conventional stimulation programs; e.g., Lotze et al., 2011; Di Stefano et al., 2012), but entitles him or her to play an active role and control the stimulation (Lancioni et al., 2010a, 2012b). The participant can determine, through the frequency of his or her responding, how much stimulation to have. More specifically, the participant can modify the response frequency in relation to his or her general status/need and also in relation to the type of stimulation available. Eventually, the participant may learn to use two or more microswitches simultaneously so as to develop new adaptive responses and choose between or among stimuli (Lancioni et al., 2010b). These opportunities can be considered essential in terms of activity engagement, self-determination, and basic communication (i.e., all critically beneficial components of an intervention program for these persons; Bosco et al., 2009; Lancioni et al., 2009a; Lotze et al., 2011; Schabus et al., 2011).

In conclusion, this study has extended the evidence available about the potential of a learning test based on operant principles. This test (a) might be more sensitive than behavioral scales normally used with post-coma persons with severe disorders of consciousness, and (b) can also serve as a functional intervention strategy aimed at enhancing adaptive responding and stimulation input, self-determination, and communication (i.e., essential targets in the rehabilitation process; Lancioni et al., 2010a). New research would need to extend the use of the learning test to other individuals with disorders of consciousness and compare the test outcome with the data of other diagnostic strategies, including behavioral scales and neurophysiological techniques (Kennedy, 2005). These research efforts could promote a better understanding of the employability, strengths, and limits of the test. Research might also focus on new responses and microswitches essential for applying the test with persons with different characteristics (Memarian et al., 2011; Lancioni et al., 2012a; Scherer, 2012).

## Conflict of Interest Statement

The authors declare that the research was conducted in the absence of any commercial or financial relationships that could be construed as a potential conflict of interest. The Associate Editor, Dr. Thomas Huenefeldt declares that, despite having collaborated with the author Dr. Marta Olivetti Belardinelli, the review process was handled objectively and no conflict of interest exists.

## References

[B1] Amantini A. Carrai R. Fossi S. Pinto F. Grippo A. (2011). The role of early electroclinical assessment in improving the evaluation of patients with disorders of consciousness. *Funct. Neurol.* 36 7–14 21693083 PMC3814503

[B2] Bagnato S. Boccagni C. Sant’Angelo A. Fingelkurts A. A Fingelkurts A. A. Galardi G. (2013). Emerging from an unresponsive wakefulness syndrome: brain plasticity has to cross a threshold level. *Neurosci. Behav. Rev.* 37(Pt 2) 2721–2736 10.1016/j.neurobiorev.2013.09.007 24060531

[B3] Barlow D. H. Nock M. Hersen M. (2009). *Single-Case Experimental Designs: Strategies for Studying Behavior Change* 3rd Edn. New York: Allyn & Bacon

[B4] Bekinschtein T. A. Shalom D. E. Forcato C. Herrera M. Coleman M. R. Manes F. F. (2009). Classical conditioning in the vegetative and minimally conscious state. *Nat. Neurosci.* 12 1343–1349 10.1038/nn.2391 19767746

[B5] Bodart O. Laureys S. Gosseries O. (2013). Coma and consciousness: scientific advances and practical considerations for clinicians. *Semin. Neurol.* 33 83–90 10.1055/s-0033-1348965 23888393

[B6] Boly M. (2011). Measuring the fading consciousness in the human brain. *Curr. Opin. Neurol.* 24 394–400 10.1097/WCO.0b013e328347da94 21577107

[B7] Bosco A. Lancioni G. E. Olivetti Belardinelli M. Singh N. N. O’Reilly M. F. Sigafoos J. (2009). Learning as a possible sign of non-reflective consciousness in persons with a diagnosis of vegetative state and pervasive motor disabilities. *Cogn. Process.* 10 355–359 10.1007/s10339-009-0334-3 19693553

[B8] Bosco A. Lancioni G. E. Olivetti Belardinelli M. Singh N. N. O’Reilly M. F. Sigafoos J. (2010). Vegetative state: efforts to curb misdiagnosis. *Cogn. Process.* 11 87–90 10.1007/s10339-009-0355-y 20043186

[B9] Bruno M. A. Vanhaudenhuyse A. Thibaut A. Moonen G. Laureys S. (2011). From unresponsive wakefulness to minimally conscious PLUS and functional looked-in syndromes: recent advances in our understanding of disorders of consciousness. *J. Neurol.* 258 1373–1384 10.1007/s00415-011-6114-x 21674197

[B10] Catania A. C. (2012). *Learning* 5th Edn. New York: Sloan

[B11] Cavinato M. Freo U. Ori C. Zorzi M. Tonin P. Piccione F. (2009). Post-acute P300 predicts recovery of consciousness from traumatic vegetative start. *Brain Injury* 23 973–980 10.3109/02699050903373493 19831494

[B12] Coleman M. R. Pickard J. D. (2011). Detecting residual cognitive function in disorders of consciousness. *Adv. Tech. Stand. Neurosurg.* 36 3–16 10.1007/978-3-7091-0179-7_1 21197605

[B13] Daveson B. (2010). An audit about music therapy assessments and recommendations for adult patients suspected to be in a low awareness state. *J. Music Ther.* 47 408–422 10.1093/jmt/47.4.408 21488605

[B14] Di Stefano C. Cortesi A. Masotti S. Simoncini L. Piperno R. (2012). Increased behavioural responsiveness with complex stimulation in VS and MCS: preliminary results. *Brain Injury* 26 1250–1256 10.3109/02699052.2012.667588 22616735

[B15] Doig E. J. Lane-Brown A. T. (2012). Responsiveness of instruments to assess disorders of consciousness: a literature review. *Brain Impair.* 13 285–315 10.1017/BrImp.2012.29

[B16] Fingelkurts A. A. Fingelkurts A. A. Bagnato S. Boccagni C. Galardi G. (2012). Toward operational architectonics of consciousness: basic evidence from patients with severe cerebral injuries. *Cogn. Process.* 13 111–131 10.1007/s10339-011-0416-x 21984310

[B17] Fingelkurts A. A. Fingelkurts A. A. Bagnato S. Boccagni C. Galardi G. (2013). The value of spontaneous EEG oscillations in distinguishing patients in vegetative and minimally conscious states. *Suppl. Clin. Neurophysiol.* 62 81–99 10.1016/B978-0-7020-5307-8.00005-3 24053033

[B18] Fischer C. Dailler F. Morlet D. (2008). Novelty P3 elicited by the subject’s own name in comatose patients. *Clin. Neurophysiol.* 119 2224–2230 10.1016/j.clinph.2008.03.035 18760663

[B19] Fischer C. Luaute J. Morlet D. (2010). Event-related potentials (MMN and novelty P3) in permanent vegetative or minimally conscious states. *Clin. Neurophysiol.* 121 1032–1042 10.1016/j.clinph.2010.02.005 20202899

[B20] Godbolt A. K. Stenson S. Winberg M. Tengvar C. (2012). Disorders of consciousness: preliminary data supports added value of extended behavioral assessment. *Brain Injury* 26 188–193 10.3109/02699052.2011.648708 22360525

[B21] Gosseries O. Bruno M. A. Chatelle C. Vanhaudenhuyse A. Schnakers C. Soddu A. (2011). Disorders of consciousness: What’s in a name? *Neurorehabilitation* 28 3–14 10.3233/NRE-2011-0625 21335671

[B22] Harrison A. H. Connolly J. F. (2013). Finding a way in: a review and practical evaluation of fMRI and EEG for detection and assessment in disorders of consciousness. *Neurosci. Biobehav. Rev.* 37 1403–1419 10.1016/j.neubiorev.2013.05.004 23680699

[B23] Hirschberg R. Giacino J. T. (2011). The vegetative and minimally conscious states: diagnosis, prognosis and treatment. *Neurol. Clin.* 29 773–786 10.1016/j.ncl.2011.07.009 22032660

[B24] Höller Y. Bergmann J. Kronbichler M. Crone J. S. Schmid E. V. Golaszewski S. (2011). Preserved oscillatory response but lack of mismatch negativity in patients with disorders of consciousness. *Clin. Neurophysiol.* 122 1744–1754 10.1016/j.clinph.2011.02.009 21377413

[B25] Kalmar K. Giacino J. T. (2005). The JFK coma recovery scale-revised. *Neuropsychol.**Rehabil.* 15 454–460 10.1080/09602010443000425 16350986

[B26] Kazdin A. E. (2001). *Behavior Modification in Applied Settings*, 6th Edn. New York: Wadsworth

[B27] Kennedy C. (2005). *Single Case Designs for Educational Research*. New York: Allyn & Bacon

[B28] Kim E. J. Park J. M. Kim W. H. Lee K. L. Kim H. N. Lee K. E. (2012). A learning set up for detecting minimally conscious state (MCS). *Ann. Rehabil. Med.* 36 428–431 10.5535/arm.2012.36.3.428 22837983 PMC3400887

[B29] King J. R. Faugeras F. Gramfort A. Schurger A. El Karoul I. Sitt J. D. (2013). Single-trial decoding of auditory novelty responses facilitates the detection of residual consciousness. *Neuroimage* 83C 726–738 10.1016/j.neuroimage.2013.07.013 23859924 PMC5635957

[B30] Lancioni G. E. O’Reilly M. F. Singh N. N. Buonocunto F. Sacco V. Colonna F. (2009a). Evaluation of technology-assisted learning setups for undertaking assessment and providing intervention to persons with a diagnosis of vegetative state. *Dev. Neurorehabil.* 12 411–420 10.3109/17518420903200581 20205550

[B31] Lancioni G. E. Singh N. N. O’Reilly M. F. Sigafoos J. Buonocunto F. Sacco V. (2009b). A technology-assisted learning setup as assessment supplement for three persons with a diagnosis of post-coma vegetative state and pervasive motor impairment. *Res. Dev. Disabil.* 30 1034–1043 10.1016/j.ridd.2009.02.006 19285830

[B32] Lancioni G. E. Bosco A. Olivetti Belardinelli M. Singh N. N. O’Reilly M. F. Sigafoos J. (2010a). An overview of intervention options for promoting adaptive behavior of persons with acquired brain injury and minimally conscious state. *Res. Dev. Disabil.* 31 1121–1134 10.1016/j.ridd.2010.06.019 20663643

[B33] Lancioni G. E. O’Reilly M. F. Singh N. N. Buonocunto F. Sacco V. Colonna F. (2010b). Post-coma persons with minimal consciousness and motor disabilities learn to use assistive communication technology to seek environmental stimulation. *J. Dev. Phys. Disabil.* 22 119–129 10.1007/s10882-009-9163-7

[B34] Lancioni G. E. Singh N. N. O’Reilly M. F. Olivetti Belardinelli M. De Tommaso M. Navarro J. (2011). A learning assessment procedure as a test supplement for monitoring progress with two post-coma persons with a diagnosis of vegetative state. *Dev. Neurorehabil.* 14 358–365 10.3109/17518423.2011.605076 21950340

[B35] Lancioni G. E. Sigafoos J. O’Reilly M. F. Singh N. N. (2012a). *Assistive Technology: Interventions for Individuals with Severe/Profound and Multiple Disabilities*. New York: Springer

[B36] Lancioni G. E. Singh N. N. O’Reilly M. F. Sigafoos J. Amenduni M. T. Navarro J. (2012b). Microswitch technology and contingent stimulation to promote adaptive engagement in persons with minimally conscious state: a case evaluation. *Cogn. Process.* 13 133–137 10.1007/s10339-011-0428-6 22131129

[B37] Lancioni G. E. Singh N. N. O’Reilly M. F. Sigafoos J. Ricci I. Buonocunto F. (2012c). Access to environmental stimulation via eyelid responses for persons with acquired brain injury and multiple disabilities: a new microswitch arrangement. *Percept. Mot. Skills* 114 353–362 10.2466/15.27.PMS.114.2.353-362 22755440

[B38] Lancioni G. E. Singh N. N. O’Reilly M. F. Sigafoos J. Alberti G. Bellini D. (2013). Persons with multiple disabilities use forehead and smile responses to access or choose among technology-aided stimulation events. *Res. Dev. Disabil.* 34 1749–1757 10.1016/j.ridd.2013.02.019 23500169

[B39] La Porta F. Caselli S. Ianes A. B. Cameli O. Lino M. Piperno R. (2013). Can we scientifically and reliably measure the level of consciousness in vegetative and minimally conscious states? Rasch analysis of the coma recovery scale-revised. *Arch. Phys. Med. Rehabil.* 94 527–535 10.1016/j.apmr.2012.09.035 23127303

[B40] Laureys S. Boly M. (2012). Unresponsive wakefulness syndrome. *Arch. Ital. Biol.* 150 31–35 23165868 10.4449/aib.v150i2.1407

[B41] Lechinger J. Bothe K. Pichler G. Michitsch G. Donis J. Klimesch W. (2013). CRS-R score in disorders of consciousness is strongly related to spectral EEG at rest. *J. Neurol.* 260 2348–2356 10.1007/s00415-013-6982-3 23765089

[B42] Lehembre R. Gosseries O. Lugo Z. Jedidi Z. Chatelle C. Sadzot B. (2012). Electrophysiological investigations of brain function in coma, vegetative and minimally conscious patients. *Arch. Ital. Biol.* 150 122–139 10.4449/aib.v150i2.1374 23165873

[B43] Lotze M. Schertel K. Birbaumer N. Kotchoubey B. (2011). A long-term intensive behavioral treatment study in patients with persistent vegetative state or minimally conscious state. *J. Rehabil. Med.* 43 230–236 10.2340/16501977-0653 21305239

[B44] Machado C. Korein J. (2009). Persistent vegetative and minimally conscious states. *Rev. Neurosci.* 20 203–220 10.1515/REVNEURO.2009.20.3-4.203 20157991

[B45] Mashour G. A. Avidan M. S. (2013). Capturing covert consciousness. *Lancet* 381 271–272 10.1016/S0140-6736(13)60094-X 23351798

[B46] Memarian N. Venetsanopoulos A. N Chau T. (2011). Client-centred development of an infrared thermal access switch for a young adult with severe quadriplegic cerebral palsy. *Disabil. Rehabil. Assist. Technol.* 6 179–187 10.3109/17483107.2010.498075 20569118

[B47] Monti M. M. (2012). Cognition in the vegetative state. *Annu. Rev. Clin. Psychol.* 8 431–454 10.1146/annurev-clinpsy-032511-143050 22224835

[B48] Monti M. M. Vanhaudenhuyse A. Coleman M. R. Boly M. Pickard J. D. Tshibanda L. (2010). Willful modulation of brain activity in disorders of consciousness. *New Eng. J. Med.* 362 579–589 10.1056/NEJMoa0905370 20130250

[B49] Nï Lochlainn M. Gubbins S. Connolly S. Reilly R. B. (2013). The vegetative and minimally conscious states: a review of the literature and preliminary survey of prevalence in Ireland. *Ir. J. Med. Sci.* 182 7–15 10.1007/s11845-012-0825-6 22528253

[B50] Owen A. M. (2013). Detecting consciousness: a unique role for neuroimaging. *Annu. Rev. Psychol.* 64 109–133 10.1146/annurev-psych-113011-143729 23043305

[B51] Owen A. M. Coleman M. R. (2008). Detecting awareness in the vegetative state. *Ann. N.Y. Acad. Sci.* 1129 130–138 10.1196/annals.1417.018 18591475

[B52] Owen A. M. Coleman M. R. Boly M. Davis M. H. Laureys S. Pickard J. D. (2006). Detecting awareness in the vegetative state. *Science* 313 1402 10.1126/science.1130197 16959998

[B53] Pierce W. D. Cheney C. D. (2008). *Behavior Analysis and Learning* 4th Edn. New York: Psychology Press

[B54] Qin P. Di H. Yan X. Yu S. Yu D. Laureys S. (2008). Mismatch negativity to the patient’s own name in chronic disorders of consciousness. *Neurosci. Lett.* 448 24–28 10.1016/j.neulet.2008.10.029 18938213

[B55] Schabus M. Pelikan C. Chwala-Schiegel N. Weilhart K. Roehm D. Donis J. (2011). Oscillatory brain activity in vegetative and minimally conscious state during a sentence comprehension task. *Funct. Neurol.* 26 31–36 21693086 PMC3814508

[B56] Scherer M. J. (2012). *Assistive Technologies and Other Supports for People with Brain Impairments*. New York: Springer

[B57] Schnakers C. (2012). Clinical assessment of patients with disorders of consciousness. *Arch. Ital. Biol.* 150 36–43 10.4449/aib.v150i2.1371 23165869

[B58] Schnakers C. Vanhaudenhuyse A. Giacino J. Ventura M. Boly M. Majerus S. (2009). Diagnostic accuracy of the vegetative and minimally conscious state: clinical consensus versus standardized neurobehavioral assessment. *BMC Neurol.* 9:35 10.1186/1471-2377-9-35 PMC2718857 19622138

[B59] Shiel A. Horn S. Wilson B. A. McLellan D. L. Watson M. Campbell M. (2000). The Wessex head injury matrix main scale: a preliminary report on a scale to assess and monitor patients’ recovery after severe head injury. *Clin. Rehabil.* 14 408–416 10.1191/0269215500cr326oa 10945425

[B60] Siegel S. Castellan N. J. (1988). *Nonparametric Statistics,* 2nd Edn. New York: McGraw-Hill

[B61] Spivey M. (2007). *The Continuity of Mind*. London: Oxford University Press

[B62] Vanhaudenhuyse A. Laureys S. Perrin F. (2008). Cognitive event-related potentials in comatose and post-comatose states. *Neurocrit. Care* 8 262–270 10.1007/s12028-007-9016-0 17990124

[B63] Vul E. Pashler H. (2012). Voodoo and circularity errors. *Neuroimage* 62 945–948 10.1016/j.neuroimage.2012.01.027 22270348

[B64] Whyte J. Gosseries O. Chervoneva I. DiPasquale M. C. Giacino J. Kalmar K. (2009). Predictors of short-term outcome in brain-injured patients with disorders of consciousness. *Prog. Brain Res.* 177 63–72 10.1016/S0079-6123(09)17706-3 19818895

[B65] Wijnen V. J. Van Boxtel G. J. Ellander H. J De Gelder B. (2007). Mismatch negativity predicts recovery from the vegetative state. *Clin. Neurophysiol*. 118 597–605 10.1016/j.clinph.2006.11.020 17239656

